# Spring-assisted posterior vault expansion—a single-centre experience of 200 cases

**DOI:** 10.1007/s00381-021-05330-5

**Published:** 2021-09-23

**Authors:** R. William F. Breakey, Lara S. van de Lande, Jai Sidpra, Paul M. Knoops, Alessandro Borghi, Justine O’Hara, Juling Ong, Greg James, Richard Hayward, Silvia Schievano, David J. Dunaway, N ul Owase Jeelani

**Affiliations:** 1 UCL Great Ormond Street Institute of Child Health & Craniofacial Unit, Great Ormond Street Hospital for Children, London, UK; 2 Paediatric Neurosurgeon, Great Ormond Street Hospital for Children, Craniofacial Unit, Great Ormond Street, London, WC1N 3JH UK

**Keywords:** Craniosynostosis, Posterior vault expansion, Spring surgery, Craniofacial surgery, Clinical outcomes

## Abstract

**Purpose:**

Children affected by premature fusion of the cranial sutures due to craniosynostosis can present with raised intracranial pressure and (turri)brachycephalic head shapes that require surgical treatment. Spring-assisted posterior vault expansion (SA-PVE) is the surgical technique of choice at Great Ormond Street Hospital for Children (GOSH), London, UK. This study aims to report the SA-PVE clinical experience of GOSH to date.

**Methods:**

A retrospective review was carried out including all SA-PVE cases performed at GOSH between 2008 and 2020. Demographic and clinical data were recorded including genetic diagnosis, craniofacial surgical history, surgical indication and assessment, age at time of surgery (spring insertion and removal), operative time, in-patient stay, blood transfusion requirements, additional/secondary (cranio)facial procedures, and complications.

**Results:**

Between 2008 and 2020, 200 SA-PVEs were undertaken in 184 patients (61% male). The study population consisted of patients affected by syndromic (65%) and non-syndromic disorders. Concerns regarding raised intracranial pressure were the surgical driver in 75% of the cases, with the remainder operated for shape correction. Median age for SA-PVE was 19 months (range, 2–131). Average operative time for first SA-PVE was 150 min and 87 for spring removal. Median in-patient stay was 3 nights, and 88 patients received a mean of 204.4 ml of blood transfusion at time of spring insertion. A single SA-PVE sufficed in 156 patients (85%) to date (26 springs still in situ at time of this analysis); 16 patients underwent repeat SA-PVE, whilst 12 underwent rigid redo. A second SA-PVE was needed in significantly more cases when the first SA-PVE was performed before age 1 year. Complications occurred in 26 patients with a total of 32 events, including one death. Forty-one patients underwent fronto-orbital remodelling at spring removal and 22 required additional cranio(maxillo)facial procedures.

**Conclusions:**

Spring-assisted posterior vault expansion is a safe, efficient, and effective procedure based on our 12-year experience. Those that are treated early in life might require a repeat SA-PVE. Long-term follow-up is recommended as some would require additional craniomaxillofacial correction later in life.

## Introduction

Children with syndromic or multi-sutural craniosynostosis often present with craniocerebral disproportion and venous hypertension and are predisposed to an underdeveloped, small posterior cranial fossa due to the prematurely fusion of the cranial sutures [1–3]. These patients are at risk of developing raised intracranial pressure (ICP), hydrocephalus, or a Chiari type 1 malformation [2–4]. To treat these problems, cranial vault expansion was traditionally undertaken via the anterior route [5, 6] with fronto-orbital advancement (FOA). However, this technique was prone to relapse, with a mean reoperation rate of 8.2%, raising to 16.7% in Apert syndrome, and higher rates of reoperation reported if FOA was performed before 6 months of age [7]. In addition, the rate of complications seen in FOA increased when subsequent frontofacial surgery was undertaken in these children, as is often required. To overcome these problems, the Birmingham Craniofacial Team (UK) introduced the posterior route for expansion of the calvarium in 1996 [8]. This approach became increasingly favoured among craniofacial surgeons as it avoids disturbance of the fronto-orbital region in the event that a subsequent frontofacial procedure is required. Posterior vault expansion (PVE) was reported to deliver greater increase in intracranial volume and, in patients with Chiari malformation, was suggested to avoid the need for foramen magnum decompression [5, 9, 10]. Rigid distraction posed challenges with skin closure, which incentivised the introduction of gradual expansion devices to support PVE. These are either transcutaneous distractors—first described by White et al. in 2009 [11]—which utilise distraction osteogenesis, or subcutaneous springs—described by Lauritzen et al. in 1998 [12]—which cause primary distraction and secondary osteogenesis [13]. Spring-assisted PVE (SA-PVE) has been the surgical technique of choice at the Craniofacial Unit, Great Ormond Street Hospital for Children (GOSH), London, UK, since 2008. This paper aims to review retrospectively the first 200 consecutive SA-PVE procedures performed at GOSH, and report the clinical experience and outcomes.

## Materials and methods

### SA-PVE operative technique

The anaesthetised patient is placed in the prone position, with the neck in a neutral or slightly flexed position. Care is taken to ensure that the abdomen is freely suspended by placing gel pads below the pelvis and the chest, to reduce central venous pressure. A single ‘Alice band’ bicoronal incision is made down to the subgaleal plane. The skin flap is reflected posteriorly to a varying degree depending on the position of the emissary veins and the subcutaneous venous anatomy.

**Fig. 1:**
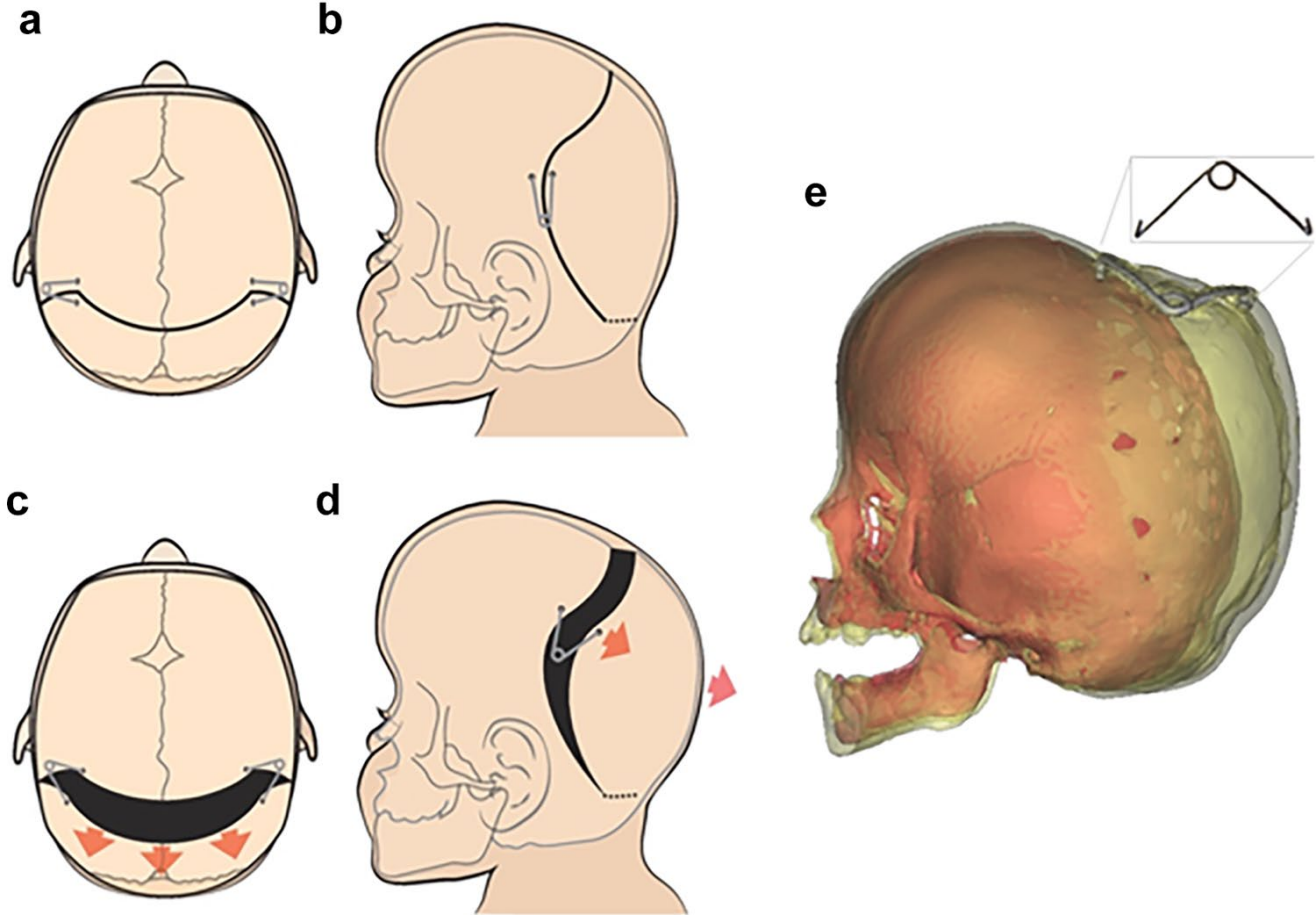
SA-PVE operative technique. Operative technique demonstrating the curved bucket handle osteotomy in axial and sagittal views (**a** and **b**), and the osteotomies and spring placement with resulting vectors (**c** and **d**). A 3D reconstruction showing the post-operative expansion achieved by the now fully open spring (**e**)

In children under the age of two, the flap is reflected back approximately 7 cm, finishing anterior to the confluence of the lambdoid sutures. Separately, a pericranial flap is dissected leaving the temporalis muscle in situ. A sub periosteal plane is next developed in the retromastoid area towards the foramen magnum, whilst the extracranial soft tissue over the torcula is left in situ. This plain typically ends within 2 cm of the foramen magnum. A curved or bucket handle (to avoid a post-operative bony step) bicoronal osteotomy line is marked 5 cm posterior to the skin incision and taken through the soft tissue tunnel towards the foramen magnum (Fig. 1a and b). A number of burr holes are made along the osteotomy line and into the retromastoid area, with the retromastoid burr holes being expanded into a small craniotomy allowing visualisation of the transverse sinus and freeing of the dura towards the foramen magnum under direct vision. The osteotomies are completed, and the dura stripped from the inner table a few centimetres anteriorly and posteriorly. The ‘give’ (movability) of the posterior bone flap is tested with the surgeon’s thumbs, and if felt sufficient, the springs can be placed. Should the flap remain tight, the osteotomies can be extended further towards the foramen magnum, or the dural dissection can be widened until sufficient ‘give’ is felt. In older children whose bones are less malleable, the soft tissue dissection may need to extend below the torcula and the posterior bone flap may be released entirely before being reattached to the calvarium using metal wires. This osteotomy is typically completed below the torcula which can be done above the torcula as well if the venous anatomy is unfavourable.

Once sufficient, ‘give’ is achieved typically two but occasionally more GOSH springs are placed into prepared grooves ensuring the footplate of the spring is locked into position. Springs are placed facing each other around 2 cm from either side of the midline (Fig. 1c and d). Spring strength (3 different wire diameters are available, resulting in 3 different spring stiffnesses [14]) is chosen by the operating surgeon; if two springs are felt to be insufficient, further springs can be placed along the osteotomy lines.

The pericranium is closed over the springs, providing stability to the construct and indicating how well the soft tissues will drape over the springs. Any spring protrusion or bony prominence can be overcome at this stage. The scalp is closed with absorbable sutures and the compressive help of an assistant, who ensures one hand is on the occiput and one on the forehead to avoid compression of the face and eyes. Once the skin is closed, the springs will begin their expansion over the ensuing days (Fig. 1).

Spring removal is done on a day case basis, under general anaesthesia. The patient is positioned as per the insertion and the original scar is reopened. The springs are uncovered using monopolar cautery. Generally, most of the bone gaps have ossified by this time; however, care must be taken to avoid dural breach in any unossified areas. Once the springs are exposed, they are removed with a combination of Mitchel’s trimmers, a Tessier periosteal elevator, and a pair of heavy forceps. Care is taken when removing the footplate not to catch any dura in the tip. The wound is closed, and no drain or dressing is used.

When repeat SA-PVE is required, the surgical technique is similar to first SA-PVE, with the additional possibility of utlising bone gaps present in the desired locations for the osteotomies. As detailed above, if not enough ‘give’ is obtained, one may need to remove the bone flap completely and resecure using sutures or steel wire, before the springs are engaged.

### Patient data and statistical analysis

A retrospective review was conducted analysing the first 200 consecutive SA-PVE performed in 184 children at GOSH—16 patients underwent a repeat SA-PVE. Data were collected from patient charts on baseline characteristics including gender, genetic diagnosis, craniofacial history before SA-PVE, indication for SA-PVE, age at time of spring insertion and removal, operative times, length of in-patient stay, transfusion requirements, complications, ophthalmology outcomes, and additional surgeries performed after SA-PVE (including repeat SA-PVE). Raised ICP was assessed by clinical findings, ophthalmology (delays in visually evoked potentials, worsening visual acuity, and papilloedema, alone or in combination), intraparenchymal pressure monitoring (ICP-bolt monitoring), and radiology evaluation, alone or in combination.

Descriptive statistics were performed to assess the study population, and different groups were compared using Student *t*-tests. Normality of the data was assessed using the Shapiro–Wilk test. Results were considered significant for *p* values < 0.05. Kaplan–Meier survival analysis with logrank testing was used to assess freedom from reintervention (repeat PVE) for patients grouped by age (0–1, 1–2, 2–4, and 4–8 years old) and diagnosis. Logrank tests were run to determine any differences in survival distribution between the age groups and diagnoses. Analysis was performed using IBM SPSS version 25 [15].

## Results

### Patient population

The first SA-PVE was performed in January 2008 with the 200th done in May 2020, in a total of 184 patients (61% male). At the time of this analysis, mean follow-up was 70 months (range, 7 months–11.8 years), 26 patients had springs still in situ, and one patient was lost to follow-up. The population consisted of 120 (65%) patients affected by syndromic and 64 (35%) by non-syndromic disorders. Underlying syndromic diagnosis was Crouzon in 35%, followed by Apert (27%), Pfeiffer (10%), Muenke (9%), Saethre-Chotzen (6%), TCF-12 (5%), ERF (3%), and 1 case each of Noonan, Smith Lemil Opitz, craniofrontonasal dysplasia, William, Bartter, Shprintzen-Goldberg, and CHARGE syndrome (Table 1). Of the 64 patients without a syndromic diagnosis, the majority (83%) included patients with multi-suture craniosynostosis without any known genetical mutation (Table 1). Repeat SA-PVE was required in 7 Apert, 4 Crouzon, 3 Pfeiffer, 1 Noonan syndrome, and 1 multi-sutural craniosynostosis.

**Table 1:** Study population demographics including diagnosis, gender, age at SA-PVE, and pre-operative craniofacial surgical history

**Diagnosis**	**Total (*n*)**	**Gender**		**No previous operations,** ***n*** **(%)**	**Median age at first SA-PVE (range, months)**
		**F**	**M**		
**Syndromic**					
**Crouzon**	42	16	26	35 (83%)	22 (5–78)
**Apert**	32	12	20	31 (97%)	13 (3–54)
**Pfeiffer**	12	9	3	8 (67%)	20 (2–83)
**Muenke**	11	6	5	10 (91%)	14 (6–59)
**Saethre-Chotzen**	7	4	3	6 (86%)	22 (9–60)
**TCF12**	6	3	3	6 (100%)	15 (9–22)
**ERF**	3	1	2	3 (100%)	53 (29–59)
**Noonan**	1	1	0	1 (100%)	2
**Smith Lemli Opitz**	1	0	1	0	85
**Williams**	1	1	0	0	23
**Bartter’s**	1	0	1	1 (100%)	48
**Sphrintzen-Goldberg**	1	0	1	1 (100%)	5
**Craniofrontonasal dysplasia**	1	0	1	1 (100%)	13
**CHARGE syndrome**	1	0	1	1 (100%)	67
*Subtotal*	*120*	53	67	104	21 (2–85)
**Non-syndromic**					
**Multi-suture synostosis**	53	13	40	47 (89%)	18 (4–131)
**Bicoronal synostosis**	6	5	1	6 (100%)	16 (8–63)
**Cranial dysraphism**	2	1	1	2 (100%)	13 (8–18)
**Sagittal synostosis**	1	0	1	0	24
**Chiari 1 malformation**	1	0	1	1 (100%)	31
**Lambdoid synostosis**	1	0	1	1 (100%)	14
*Subtotal*	*64*	19	45	57	17 (4–131)
**Total**	**184**	**72**	**112**	**161 (88%)**	**19 (2**–**131)**

Eighty-seven percent of syndromic and 89% of non-syndromic patients had no other transcranial procedures before SA-PVE (Table 1). A total of 26 (14%) patients had undergone other transcranial procedures before first SA-PVE. These included 5 anterior posterior shortening with biparietal expansions, 5 posterior cranial vault remodelling (PCVR) procedures, 3 FOA procedures, 2 foramen magnum decompression (FMD) procedures, 2 spring-assisted cranioplasties for scaphocephaly, 2 monobloc and rigid external distraction (RED) frame procedures, and 1 sagittal suturectomy. Six patients had undergone multiple transcranial procedures: one patient had a PCVR and an FMD; one had two FMD procedures; one had PCVR followed by monobloc and RED-frame, followed by anterior posterior shortening with biparietal expansions and another PCVR; one had total calvarial remodelling and a PCVR; one had two PCVR procedures; and one had anterior posterior shortening with biparietal expansions followed by FOA. One patient had FOA at the time of their SA-PVE.

SA-PVE was indicated for ICP-related concerns in 149 (75%) patients, of which 138 (93%) had confirmed raised ICP and 11 (7%) were considered to be at high risk of developing raised ICP in the near future. Decision to operate in these cases was based on literature and clinical experience. Raised ICP was confirmed by deteriorating ophthalmological findings in 87 (63%) cases; ICP-bolt monitoring in 32 (37%); worsening clinical picture indicative of raised ICP in 6 (4%); radiographic findings in 6 (4%); and a combination of the above in the rest. SA-PVE was indicated for (turri)brachycephalic head shape improvement in the remaining 35 (25%) patients. Repeat SA-PVE was performed due to raised ICP concerns in 14 patients and for head shape improvement in 2.

### SA-PVE surgeries

Median age at spring insertion for first SA-PVE was 19 months (range, 2–131 months) and at removal 34 months (range, 3–144 months), with springs remaining in situ for a mean of 9 months (range, 3 days–51 months). First SA-PVE spring insertion happened at a median age of 21 months for syndromic vs. 17 months for non-syndromic cases. Repeat SA-PVE was performed at a median age of 31 months (range, 17–93 months), with removal at 41 months (range, 23–68 months); the springs remained in situ for 10 months (range, 13 days–33 months; no statistically significant difference compared to first SA-PVE).

#### Repeat SA-PVE

**Fig. 2:**
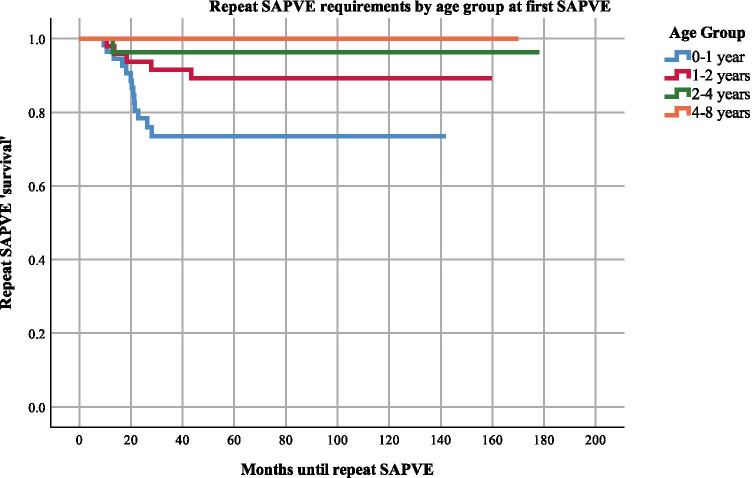
Kaplan–Meier survival analysis on time to repeat SA-PVE by age group at time of first SA-PVE. This survival analysis evaluates the likelihood that patients needed a second SA-PVE based on age information. A survival of 1.0 indicates no repeat SA-PVE. From this graph, we can learn that the zero-to-one age group was more likely to require a repeat SA-PVE

**Fig. 3:**
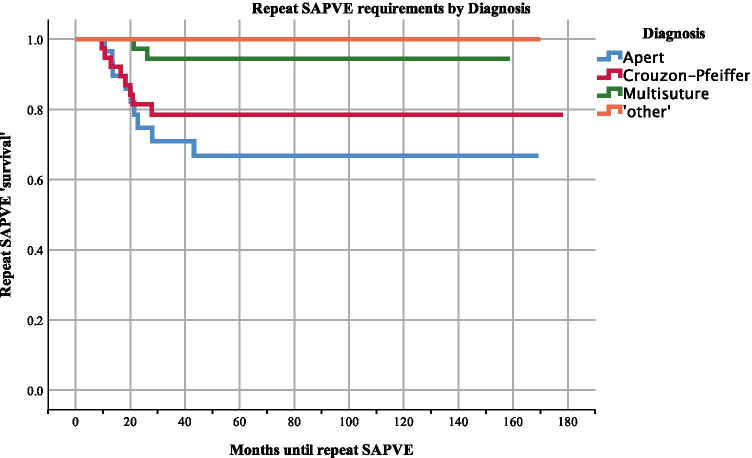
Kaplan–Meier survival analysis on time to repeat SA-PVE by diagnosis. This survival analysis evaluates the likelihood that patients needed a second SA-PVE based on diagnosis information. A survival of 1.0 indicates no repeat SA-PVE. The repeat SA-PVE was required mostly for the Apert population, followed by Crouzon-Pfeiffer, and multi-suture

Figures 2 and 3 show the Kaplan–Meier survival curves illustrating the time until repeat SA-PVE, by age group and diagnosis, respectively. Patients aged 0–1 year old showed a significant increased need for a repeat SA-PVE compared to older patients (logrank test, *p* = 0.01). There was a significant difference in the requirement for SA-PVE also between diagnostic groups (logrank test, *p* =  < 0.001). Patients with Apert syndrome, followed by Crouzon and Pfeiffer required a repeat SA-PVE more frequently compared to the rest of the diagnostic groups and at a time point closer to their initial SA-PVE than multi-sutural synostosis and ‘other’ diagnosis patients. Patients with Apert or Crouzon and Pfeiffer required repeat SA-PVE at similar ages.

#### Fronto-orbital advancement at time of spring removal

A total of 41 (26%) patients had FOA during spring removal. This group consisted of 15 patients diagnosed with multi-sutural synostosis (37% of all multi-sutural SA-PVE removals), 6 of 11 Muenke, 5 of 6 TCF-12, 3 of 7 Saethre-Chotzen, 3 of 6 isolated bicoronal synostosis, 3 of 32 Apert, 2 of 42 Crouzon, 1 of 12 Pfeiffer, and the unique cases of Bartter’s syndrome, Sphrintzen-Goldberg, and craniofrontonasal dysplasia.

Spring removal combined with FOA was performed in significantly younger patients than spring removal alone (15 months vs. 25 months, respectively, *p* = 0.005). There was no statistically significant difference between length of time of springs in situ for cases with FOA at time of spring removal (average 12 months; range, 1–41 months) or for cases without an additional FOA.

#### Operative time

The mean operative time for insertion of springs without additional procedures was 2 h 30 min (range, 1–5 h 35 min) and for removal was significantly shorter at 1 h and 26 min (range, 32 min–4 h and 10 min, *p* =  < 0.001). When removal of springs was combined with FOA, the mean operative time was significantly longer, at 3 h and 11 min (range, 1 h and 8 min–4 h and 45 min, *p* =  < 0.001).

### Post-SA-PVE and clinical outcomes

#### Hospital stay

The median in-patient hospital stay for SA-PVE insertion was 3 nights (range, 1–116 nights); the median stay for removal alone was 1 night (range, 0–160 nights). When removal of springs was combined with FOA, the median stay was 4 nights (range, 1–51 nights), with no statistically significant difference from stay after spring insertion.

#### Ophthalmology

Ophthalmology data post-surgery were available for 102 patients. An overall post-operative improvement was seen in either visual acuity or papilloedema in 99 patients (97%). Of the three patients that showed no improvement, one had stable visual acuity that showed no worsening, and two had worsening acuity and no improvement in papilloedema.

#### Transfusion requirements

At first SA-PVE, 122 patients received a mean of 196 ml of allogenic blood. For spring removal in those patients not undergoing FOA at time of spring removal, 16 patients received a mean volume of 63 ml. Of the 41 cases where spring removal was combined with FOA, 32 patients required a mean transfusion of 425 ml at removal.

#### Complication profile

Complications were assessed for all patients with the Oxford craniofacial complication scale, ordinarily used to compile complication data for the annual UK Craniofacial National Audit [16]. The scale consists of 0–5 grades as described in Table 2.

**Table 2:** Overview of SA-PVE complications using the Oxford Craniofacial Complication Scale

**Grade**	**Complication description**	**SA-PVE**				**Total**
		**1st insertion**	**1st removal**^a^	**Repeat insertion**	**Repeat removal**	
0	No complications	158	155	14	16	343
1	No delay in discharge, reoperation, or long-term sequelae	2	0	0	0	2
2	Delay in discharge but no further operation required	3	2	1	0	6
3	Reoperation but no long-term sequelae	22	0	1	0	23
4	Unexpected long-term deficit or neurological impairment (permanent disability)	0	0	0	0	0
5	Mortality	1	0	0	0	1
	Total complications of grade 1–5	28	2	2	0	32

^a^Twenty-six springs remain in situ at time of current analysis

A total of 32 complications occurred in 26 patients, of which 30 events were related to insertion of springs at the SA-PVE and 2 events were related to spring removal. We had one mortality in this series. This patient had cranial dysraphism, pansynostosis, and a history of a large vertex encephalocele treated with a VP shunt at the age of 7 weeks. The patient was referred to our unit at the age of 3 for repair of the encephalocele. The surgery was uneventful, and the patient woke up with a Glasgow Coma Scale score of 12 post-operatively. Approximately 10 h after surgery, the patient had a sustained seizure presumed to be secondary to a ventriculoperitoneal shunt malfunction. A computed tomography scan showed a ‘tight looking brain’. A bifrontal decompression was performed and the shunt externalised. Despite these manoeuvres, ICP continued to rise over the ensuing 72 h, and along with the family’s wishes, ongoing care was withdrawn.

The other 31 complications were graded between 1 and 3. Twenty-seven events were related to insertion of springs at first SA-PVE and consisted of three grade 2 events—one patient required intravenous antibiotics following a post-operative chest infection and one patient sustained a small sinus breach intraoperatively causing intra-operative bleeding, which required a post-operative transfusion. Another patient was initially treated elsewhere and presented with an unusually located scar from a previous bicoronal incision. At GOSH, a standard bicoronal incision was performed, which caused scalp flap compromise and skin necrosis between the two incisions. This resolved with non-surgical management but resulted in secondary alopecia. Grade 3 complications occurred in 19 patients, with 22 events: one subgaleal collection requiring washout; two retained drains that required removal under general anaesthetic; nine eroding or outwardly dislodged springs that required early removal; and ten post-operative surgical site infections requiring removal of springs, washout, and antibiotic treatment.

At time of spring removal after first SA-PVE, two grade 2 complications were reported: one patient had a minor bleeding post-operatively which was observed and managed conservatively; and one patient required intravenous antibiotics for a wound infection. At repeat SA-PVE, two complications occurred: one grade 2, where a patient with known central and obstructive sleep apnoea had a respiratory arrest on the ward and made a full recovery; and one grade 3, where a patient returned to the operating theatre for management of a post-operative haematoma.

#### Follow-up

A total of 26 patients underwent at least one additional craniomaxillofacial procedure after SA-PVE, of which 2 following their repeat SA-PVE. Eleven monobloc advancements with RED-frame distraction were performed in 9 patients at an average age of 45.6 months (range, 10–97). One of these patients later required FMD and a further monobloc advancement with RED-frame. Anterior 2/3 remodelling was done in four patients at an average of 10 months (range, 6.4–16.3), of which one patient had monobloc advancement with RED-frame distraction 4 years after the anterior 2/3 remodelling. Further posterior vault remodelling was performed in 4 four patients at an average age of 36.5 months (range, 28–57). Le Fort III with RED-frame distraction was performed in two patients, of which one 16 months, and one 7.7 years after SA-PVE. The latter required two further FOAs. Following repeat SA-PVE, the two patients had FMD, which was undertaken at 10- and 18-months post-repeat SA-PVE (Table 3).

**Table 3:** Overview additional craniomaxillofacial procedures following SA-PVE

	Number of additional craniomaxillofacial procedures by diagnosis			**Total**
	**Apert**	**Crouzon-Pfeiffer**	**Multi-sutural synostosis**	
Monobloc + RED-frame	2	9	0	**11**
Le Fort III + RED-frame	0	1	1	**2**
Anterior 2/3 remodelling	0	1	3	**4**
Foramen magnum decompression	0	3	0	**3**
Frontal orbital advancement	0	2	0	**2**
Posterior cranial vault remodelling	2	1	1	**4**
**Total procedures**	**4**	**17**	**4**	**26**

*RED*, rigid external distraction

## Discussion

This study reports on our experience of the first 200 consecutive SA-PVEs, delivered via a standardised surgical approach utilising spring devices [14]. This is a large clinical experience and our aim was to report our entire series utilising this surgical technique. Clinically, it is a heterogeneous group of indications for which this technique was used. The main inclusion criteria were the cohort of patients where we would have traditionally undertaken a static PVE. During the study period a small portion of children, typically over age 7 years, continued to be managed utilising the traditional approach.

SA-PVE achieved satisfactory gradual primary expansion of the skull with secondary callus ossification. The elastic nature of the spring design allows for easier and more robust soft tissue closure after implantation, thus overcoming this limitation of the more traditional PCVR. Additionally, spring action over time followed by consolidation until spring removal reduces the risks of relapse which may occur after PCVR. Compared to external distractors used in PVDO, the internal springs chosen at GOSH require no continuous break of the skin barrier, thus reducing infection risks, and no need for carers’ compliance with distraction protocols over a long period of time [17]. The forces and future perspectives on positioning on springs and surgical planning are well-described in the work of Borghi et al. [14, 18, 19]. The main disadvantages of SA-PVE compared to PCVR are the need for a second operation to remove the springs and the inability to reshape gross contour abnormalities such as the correction of flattened or hypoplastic regions or compensatory bulges. In SA-PVE, only the craniotomised segment can be advanced, but the contour of the expanded region cannot be significantly altered.

SA-PVE showed comparable trends and outcomes to traditional vault expansion procedures reported in the literature [1, 5, 9, 10]. In our series, the majority of surgeries were delivered in syndromic patients, mostly with Crouzon syndrome followed by Apert. Apert, Muenke, and TCF12 were operated earlier compared to other syndromes.

Raised ICP concerns were the predominant driver for SA-PVE in our series. The majority of raised ICP findings were diagnosed on ophthalmological findings, including a combination of visually evoked potentials, worsening visual acuity, and the finding of papilledema [20]. Although this study showed a 97% improvement of papilledema as a result of the SA-PVE, details of ophthalmology results presented in this study are limited and an in-depth study should be carried out to identify specific ophthalmologic tests and outcomes to evaluate raised ICP in children with craniosynostosis. The remainder of the study population was indicated for solely shape improvement, future studies include objective analysis of these outcomes.

A reduced level of control over the vector of the posterior distraction has been discussed as a potential disadvantage of SA-PVE compared to traditional vault expansion surgeries; this has not been the case in our series. Whilst the distraction resulted in some asymmetric for a proportion of the patients, in no cases was it significant enough to warrant any further surgical adjustment. A quantification of SA-PVE shape outcomes using 3D surface analysis as carried out for spring-assisted cranioplasty in sagittal craniosynostosis would allow us to improve further the location of the surgical osteotomies, and the spring device choice and positioning [21].

With a total average operative time (insertion and removal of springs) of approximately 4 h, the operative time for SA-PVE is comparable to PVDO that is reported with an average of 3 h and 48 min, and with SA-PVE reported from another centre, taking 3 h and 35 min. Albeit with weak correlation, the SA-PVE operative time at GOSH has decreased over the last 12 years. Moreover, the surgical time is comparable to traditional PCVR with a mean total operative time of 2 h and44 min [6, 22]. The median hospital stay lengths from the current study (3 nights) are in concordance with the literature describing SA-PVE and PVDO at 4 days and 3.25 days, respectively [6, 22]. Currently at GOSH, we are aiming for spring removal to be performed as a day case. The blood transfusion requirements in our study are in concordance with a study on 31 PVDO which reported an 80% transfusion rate, with an average of 270 ml [1].

A significantly higher number of repeat SA-PVE were required in patients who underwent their first SA-PVE earlier in life, i.e., aged 0–1 year old compared to older patients. Moreover, patients with Apert syndrome, followed by Crouzon and Pfeiffer required more repeat SA-PVE procedures than the rest of the study population. We hypothesised that the severity of both frontal and posterior cranial deformities influences the age and amount of expansion needed. Cranial vault volumetric changes are expected from SA-PVE; however, these results are a subject of another study [23].

Our series demonstrated 32 post-operative complications in 26 patients following SA-PVE. There was mortality; this patient had a complex medical history and a diagnosis of cranial dysraphism. The other complications included 23 cases with return to theatre requiring a general anaesthetic. These results are similar to the 16% described in PVDO by the Oxford Craniofacial Team [1]. Surgical site infections were the most common complications. Infections settled after spring removal, apart from two cases with osteomyelitis.

Although SA-PVE relieved the raised ICP concerns in the majority of our study population, some patients went on for further frontofacial procedures. Craniofacial craniosynostosis syndromes are characterised by complex and variable phenotypes affected with not only calvarial deformity but also midfacial anomalies. Some patients with additional midfacial hypoplasia and orbital deformity, such as Crouzon and Pfeiffer (11/54) went on for facial surgery post-SA-PVE. Also, 3/32 patients with Apert syndrome required frontofacial surgery either at time of spring removal or at adolescence for aesthetic reasons, and 2 patients further underwent a monobloc distraction [24, 25]. Given the length of follow-up, further SA-PVE and midface or frontofacial procedures are likely to be required in the included study population.

As expected, there is a learning curve with this technique. We start off by using a larger number of springs (typically 6) and over time settled with 2 springs per procedure. Our osteotomies were more extensive in the beginning with, and gradually, these became more limited with similar surgical outcomes. Out bucket handle craniotomy was modified to a more sinusoidal shape (as shown in Fig. 1) to prevent a bony lip at the top of the head. We are currently in the process of undertaking subgroup analyses from this cohort to better define these parameters and their utility in specific clinical cohorts utilising techniques used in our sagittal synostosis cohort [14, 19, 23]. These we hope to present to the surgical audience in the near future.

## Conclusion

Children with raised intracranial pressure and those with brachy(turry)cephalic head shapes can benefit from spring-assisted posterior vault expansion. Following a 12-year experience, we found this to be our surgical technique of choice with a comparable adverse event profile compared to more traditional and alternative treatment options.
